# Silanization of Cotton Fabric to Obtain Durable Hydrophobic and Oleophobic Materials

**DOI:** 10.3390/ijms262311374

**Published:** 2025-11-25

**Authors:** Anna Szymańska, Marcin Przybylak, Agnieszka Przybylska, Hieronim Maciejewski

**Affiliations:** 1 Poznań Science and Technology Park, Adam Mickiewicz University Foundation, Rubież 46, 61-612 Poznań, Poland; anna.szymanska@ppnt.poznan.pl (A.S.); hieronim.maciejewski@ppnt.poznan.pl (H.M.); 2 Faculty of Chemistry, Adam Mickiewicz University, Uniwersytetu Poznańskiego 8, 61-614 Poznań, Poland; agnieszka.przybylska@amu.edu.pl

**Keywords:** cotton fabric, hydrophobicity, oleophobicity, silanes, chemical modification

## Abstract

Developing durable hydrophobic and oleophobic textiles using simple and environmentally responsible techniques remains a challenge. This study aimed to determine how the structure of organosilicon silanes—specifically the type of functional group (fluorinated alkyl, long alkyl, or benzyl group) and the presence of an ester linker formed via the thiol–Michael addition—affects the wetting behaviour of cotton fabrics. Five silanes were synthesized and applied using a mild pad–dry–cure silanization process. The modified fabrics were evaluated through water and oil contact angle (WCA, OCA) measurements, water absorption tests, droplet-stability analysis, and washing-durability assessment. All treated samples exhibited hydrophobicity, while the silane containing a C6 perfluoroalkyl chain provided both hydrophobic and oleophobic performance. This fabric showed a WCA of 152° and an OCA of 126° (hexadecane), which remained essentially unchanged after 10 washing cycles (153° and 126°, respectively). Water absorption decreased by 91%, and droplets remained stable for at least 30 min. SEM, and SEM-EDS confirmed the presence and uniform distribution of the silane coating. These results demonstrate that short-chain fluorinated silanes and long-chain alkyl silanes can form durable low-surface-energy layers on cotton using a straightforward and efficient process, offering a promising route for high-performance functional textiles.

## 1. Introduction

For decades, materials possessing hydrophobic or oleophobic characteristics have been attractive due to their wide range of applications, including self-cleaning materials [1,2,3,4], oil–water separation [5,6,7,8], anti-corrosion coatings [9,10,11], anti-icing coatings [12,13,14] and so on, but in many cases materials can possess multifunctional applications.

The wettability of materials such as cotton fabric, which is hydrophilic and oleophilic in nature, can be modified by proper surface roughness and by chemically modifying it with low surface energy materials, for example silanes or fluorosilanes [15,16,17]. Most frequently, fluorine or silicone is combined with immobilization on fiber surface nanoparticles such as SiO_2_, ZnO, or TiO_2_ [18,19,20,21,22].

Alkyl and fluoroalkyl group-containing silanes and silicones are often used types of organosilicon compounds to obtain hydrophobic and oleophobic materials [16,23]. Fluorinated compounds are commonly used to modify the properties of materials, including cotton, due to their ability to impart both hydrophobic and oleophobic properties [24]. Oil-repellency can be achieved through combining fluorinated materials with the appropriate surface topography [25]. Perfluoroalkyl substances (PFAS) are classified depending on the number of fully perfluorinated carbon atoms in the chain, falling into three categories: ultra-short-chain (from C2 to C3), short-chain (from C4 to C7) and long-chain (from C8 to C14) [26]. Replacing fluorine atoms with hydrogen and carbon causes an increase in surface free energy. The surface free energy is decreased as follows: CH_2_ > CH_3_ > CF_2_ > CF_2_H > CF_3_ [27]. The longer the fluorinated alkyl chains, the lower surface energy and the higher the static contact angle [28]. However, some compromise must be made, because longer carbon chains of perfluorinated compounds mean greater toxicity and bioaccumulation. As a result, restrictions have been introduced on the use of fluoropolymers/fluorotelomers having fluorinated side chains with C8 or longer and it is therefore necessary to look for solutions to eliminate compounds containing fluorine [18].

To obtain oleophobicity the key is to achieve the highest possible concentration of CF_3_ groups on the surface of the material and stabilize the surface. Longer chains form more crystalline, densely packed chains, with a high concentration of CF_3_ moieties on the surface, while shorter chains do not form highly crystalline layers which limits the density of surface CF_3_ groups and limits oil repellency [26]. Park et al. have demonstrated that dynamically oleophobic surface can be achieved without using long-chain PFAS and surface roughening. They prepared a dynamical oleophobicity coating by sol–gel method using short-chain fluorinated silane 3,3,3-trifluoropropyltrimethoxysilane and tetramethoxysilane (TMOS). TMOS were used as spacer to increase the physical mobility of functional groups tethered to the surface [29]. Incorporation of spacer between fluorinated chain and the polymer can allow the self-arrangement of the fluorinated chains [30]. Wetting behavior and molecular motion depend on α-substituent and spacer between the polymer backbone and the perfluorinated groups of the side chains [30,31,32,33,34,35,36]. Wang et al. have synthesized polymer film on cotton fabric formed of long-chain stearyl acrylate and short-chain perfluorohexylethyl methacrylate. In their research, water repellency of the coated cotton fabric was tested, with both experimental and computer simulation studies confirming that surface energy is not a decisive factor in determining hydrophobicity [37]. Tan et al. have synthesized the short fluoroalkyl copolymer anchored with a short perfluoroalkyl group (C6), various long hydrocarbon alkyl side chain (−C_n_H_2n+1_, *n* = 12, 14, 16, 18), and hydroxyethyl methacrylate. When *n* = 18, coated fabric exhibits superhydrophobic and oleophobic properties, with a WCA of 150.5 ± 0.5° and a hexadecane CA of 116.1 ± 0.2° [36]. Li et al. have grafted fluorinated polyhedral oligomeric silsesquioxane (POSS) through silane coupling of 1*H*,1*H*,2*H*,2*H*-perfluorodecyltrimethoxysilane and 3-mercaptopropyltriethoxysilane (MPTES) and thiol-ene click reaction between octavinyloctasilasesquioxane, MPTES and 1*H*,1*H*,2*H*,2*H*-perfluorododecyl-1-thiol. The modified cotton fabrics exhibited superhydrophobicity and oleophobicity with WCA of 163.5° and OCA of 120.1°, longer perfluoroalkyl groups (chain of eight fluorinated carbon atoms) were applied [38].

The hydrophobicity of textile materials is much easier to achieve than oleophobicity. In the case of the latter, the challenge is to obtain an oil-repellent coating without using fluorinated compounds, whereas in the case of hydrophobicity, the range of possibilities is much wider. One of the options is to apply fluorine-free silicone-based compounds. Examples of such groups of compounds include polydimethylsiloxanes, long alkyl chain silanes, long alkyl chain polymers, and polyhedral oligomeric silsesquioxane, etc. [39,40,41,42].

Although silane derivatives are widely used for surface modification of textiles due to their versatility, strong adhesion to hydroxy group at substrates, and ability to impart hydrophobic or oleophobic properties, they also present certain limitations. The effectiveness of silane-based coatings strongly depends on precise control of hydrolysis and condensation reactions, which can affect the uniformity and durability of the deposited layer. Moreover, some fluorinated silanes are associated with environmental and health concerns, motivating the search for safer alternatives. In the textile field, silane-based modifications have shown promise in producing water- and oil-repellent, self-cleaning, and protective fabrics. However, developing stable, durable, and eco-friendly coatings that maintain fabric flexibility and breathability remains a key challenge and the focus of ongoing research.

The main objective of this study was to develop a simple, efficient, and durable silanization method for modifying cotton fabrics to achieve both hydrophobic and oleophobic surfaces. The research focused on understanding how the structure of silane derivatives—particularly the type of functional group, the length of the fluorinated chain, and the presence of an ester linker formed via the thiol–Michael reaction—influences the wetting behavior of the treated fabrics. The silane modifiers, previously synthesized in our earlier work, were applied to cotton under mild conditions to ensure strong chemical bonding with surface hydroxy groups [43]. The modified fabrics were thoroughly characterized using SEM, and SEM-EDS analyses to confirm the presence and distribution of the modifiers. Their hydrophobic and oleophobic performance, washing durability, and potential for practical textile applications were evaluated through contact angle measurements, water absorption tests, and droplet stability observations. The results demonstrate that it is possible to obtain durable fabrics exhibiting both hydrophobic and oleophobic characteristics, highlighting the applicability of this approach for advanced textile finishes with self-cleaning properties and resistance to various stains.

## 2. Results

### 2.1. Preparation of Cotton Fabric Samples

In our previous publication [43], we reviewed initiators and catalysts for the thiol-Michael addition reaction and then obtained several new organosilicon compounds. Four compounds were selected (S1–S4) and used for the modification of cotton fabrics so that the products obtained via the thiol-Michael addition reaction allowed the fabrics to obtain hydrophobic or oleophobic properties. Additionally, a fifth silane (S5) was synthesized using the thiol-ene reaction The compound was synthesized in an easy and quick way, via the addition of SH group of 1-dodecanethiol to a HC=CH2 bond of trimethoxyvinylsilane in the presence of a photoinitiator (2,2-dimethoxy-2-phenylacetophenone, DMPA) and UV light. The structure of the synthesized compound was confirmed by NMR spectroscopy. The product obtained with 81% isolation yield (1H, 13C and 29Si NMR spectra are included in Supplementary Information (Figures S1–S3)). The obtained derivative was applied to the cotton fabric, differing in its linker from the others. The structural formulas of the silanes used are shown in Table 1.

**Figure 1:**
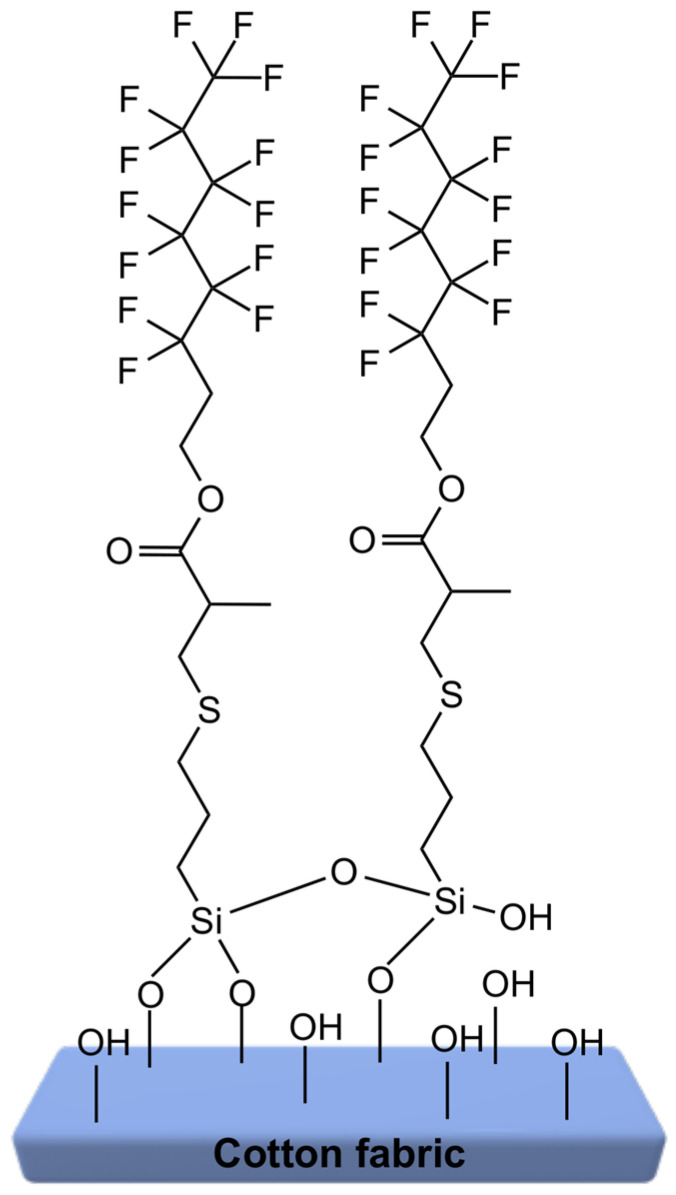
Proposed schematic illustration how to bind and orient silane S2 on the surface of cotton.

Silanes are composed of two functional groups: an alkoxysilyl group, which reacts with the hydroxyl groups of cotton fibers through hydrolysis and condensation, and a second functional group responsible for imparting hydrophobic or oleophobic properties. Between these two groups, an ester linker is present, formed via the thiol–Michael addition between thiols and (meth)acrylates. This ester moiety can affect the molecular arrangement of the modifier on the cotton surface by enabling dipole–dipole interactions between the carbonyl groups of neighboring molecules. Such interactions may promote a more ordered orientation of the perfluoroalkyl chains on the fiber surface, leading to a higher surface density of CF_3_ groups and, consequently, enhanced water and oil repellency. Sample S2 was selected to illustrate the proposed mechanism of silane bonding to the cotton surface, as this silane provided the most favorable results in terms of hydrophobic and oleophobic performance, showing the highest contact angle values and best surface stability among all the tested modifications (Figure 1).

### 2.2. Add-On and SEM-EDS Results

The add-on values and results of the SEM–EDS analysis of the modified samples before and after the washing process are presented in Table 2.

The add-on values indicative of the amount of silane retained by the fabric varied depending on the silane applied. It was observed that samples modified with fluorinated silanes (CS1 and CS2) exhibited lower add-on percentages compared to those modified with non-fluorinated counterparts (CS3 and CS4). This result can be attributed to the inherently lower surface energy of fluorinated compounds, which limits their affinity and interaction with hydrophilic cotton fibers, consequently resulting in reduced deposition.

It is important to highlight that repeated washing cycles did not significantly decrease the add-on values of the modified fabrics, demonstrating the excellent durability and wash resistance of the applied modification.

The elemental composition of the impregnated fabrics was tested in order to confirm the modification. SEM-EDS analysis provided further insight into the chemical composition of the modified fabrics, corroborating the add-on results. Unmodified cotton exhibited the typical composition dominated by carbon (approximately 34 wt.%) and oxygen (around 64 wt.%), with no detectable silicon, sulfur, or fluorine. After modification, SEM-EDS revealed clear signals of silicon and sulfur, (and fluorine for CS1 and CS2), confirming the successful grafting of silane-based modifiers onto the cellulose fibers. Samples CS1 and CS2, treated with fluorinated silanes, showed lower silicon and sulfur content compared to samples CS3 and CS4, modified with non-fluorinated ones. This aligns well with the measured add-on values, where fluorinated silanes exhibited lower deposition levels, likely due to their inherently lower affinity toward the hydrophilic cellulose substrate.

After subjecting the modified fabrics to multiple washing cycles, SEM-EDS data indicated minimal changes in the elemental composition, demonstrating the durability of the silane treatments. The retention of Si, S, and F elements post-washing suggests that the applied modifications are resistant to laundering, maintaining their functional properties over time. This durability is crucial for practical applications, as it ensures the longevity of the fabric’s enhanced characteristics.

The SEM-EDS analysis corroborates the add-on values, confirming the successful and durable modification of cotton fabrics with silanes S1 through S4. The findings highlight the influence of silane composition on deposition efficiency and underscore the effectiveness of these treatments in producing fabrics with sustained functional properties.

### 2.3. Elasticity Measurement Results

The results showed (Table 3) that the silane-treated fabrics demonstrated bending lengths and flexural rigidity values very similar to those of the untreated cotton. This indicates that the thin, low-add-on silane layers—formed under mild hydrolysis and curing conditions—did not stiffen the fabric or negatively affect its mechanical flexibility. Importantly, the flexibility of the fabrics remained unchanged after ten washing cycles, confirming both the durability. Maintaining fabric softness and drape is essential for clothing, upholstery, and technical textile applications, and the applied modification method successfully preserves these desirable tactile characteristics.

### 2.4. SEM Images

**Figure 2:**
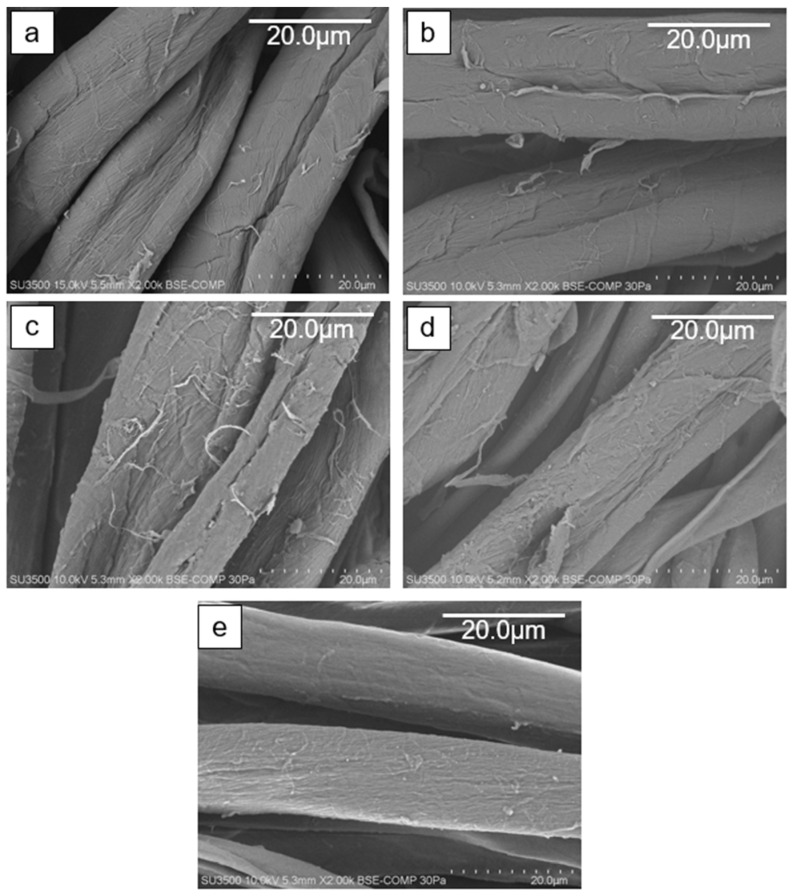
SEM images of unmodified cotton (**a**), and modified CS1 (**b**), CS2 (**c**), CS3 (**d**), and CS4 (**e**) cotton fabrics.

SEM images of raw cotton and modified cotton fabrics (CS1-CS4) are presented in Figure 2.

The surface of the raw cotton, as well as all the modified cotton fabrics, are smooth, without aggregates or visible coating film. This indicates a subtle modification of fabrics without disturbing its structure or causing roughness.

### 2.5. Hydrophobic Properties of the Modified Cotton Fabrics

**Figure 3:**
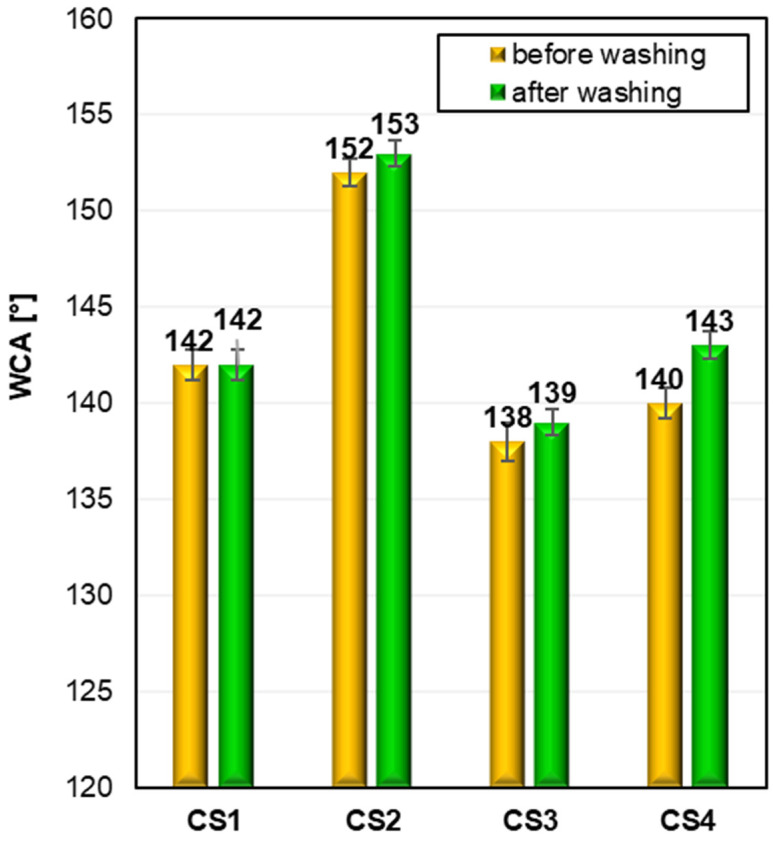
Water contact angle (WCA) of modified cotton fabrics (CS1–CS4) before and after washing. The corresponding standard deviations for samples are as follows: CS1 142 ± 0.8, 142 ± 0.8; CS2 152 ± 0.7, 153 ± 0.7; CS3 138 ± 1, 130 ± 0.7; CS4 140 ± 0.8, 143 ± 0.7.

To determine the surface properties of the modified cotton fabrics, the water contact angle (WCA) was measured. Each value represents the mean of five independent measurements. The measured WCA values of the modified cotton fabrics before and after washing process are shown in Figure 3.

The raw cotton fabric has hydrophilic properties, and it is not possible to measure the WCA value because a drop of water absorbs immediately. All silane-modified cotton samples are hydrophobic. The WCA measurements revealed notable differences depending on the type of functional group present in the silane modifiers used. The most effective hydrophobic performance was obtained using the silane featuring a long-chain fluorinated substituent (CS2), resulting in cotton fabrics exhibiting superhydrophobic characteristics with a remarkable WCA value of approximately 153°. However, it is important to note that the mere presence of fluorine atoms in the molecular structure did not automatically ensure superior hydrophobicity, as illustrated by the branched fluorinated silane (CS1), which showed comparable WCA values to those obtained from the non-fluorinated silane bearing a long alkyl chain (CS4). This indicates that molecular geometry and the arrangement of functional groups also significantly influence the hydrophobic outcome. In contrast, the lowest hydrophobic performance was observed for the silane bearing a benzyl substituent (CS3). Despite exhibiting the lowest WCA values among the tested silanes, this aromatic derivative nonetheless imparted high hydrophobicity to the cotton surface, confirming the effectiveness of the silane modification in general. Overall, the study demonstrates that the type and structure of substituents strongly impact the achieved WCA, and both fluorinated and non-fluorinated alkyl silanes can yield comparably high levels of hydrophobicity.

**Figure 4:**
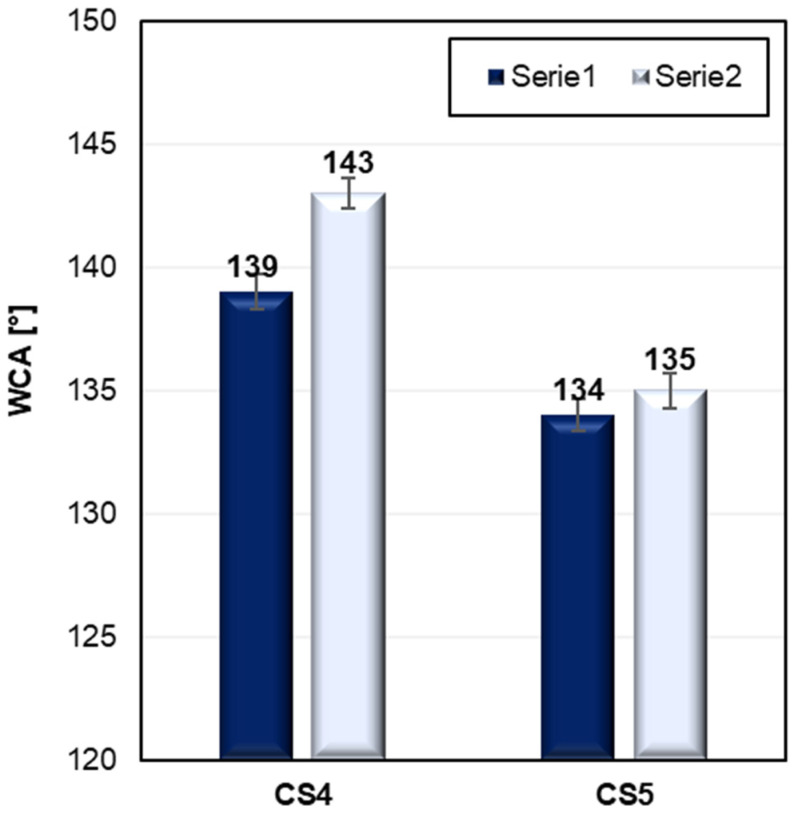
Water contact angle (WCA) of modified cotton fabrics CS4 and CS5 before and after washing. Comparison of silanes S4 and S5. The corresponding standard deviations for samples are as follows: CS4 139 ± 0.7, 143 ± 0.6; CS5 134 ± 0.6, 135 ± 0.7.

To verify our hypothesis regarding the role of ester linkages in the structure of silanes in enhancing hydrophobicity, an additional silane derivative (S5) was synthesized, featuring an alkyl chain of identical length to S4 but lacking the ester linkage. WCA values of modified cotton fabrics CS4 and CS5 before and after washing are shown in Figure 4.

Comparative tests revealed that fabrics modified with silane S5 consistently exhibited lower water contact angles than those treated with silane S4 (142° vs. 134° for unwashed samples, and 142° vs. 135° for washed ones). These results support our assumption that the presence of an ester group between two functional groups plays a beneficial role in promoting surface hydrophobicity.

One possible explanation for this effect lies in the increased conformational flexibility imparted by the ester moiety, which facilitates better molecular rearrangement and orientation of functional groups that provide hydrophobic properties. Furthermore, dipole–dipole interactions between adjacent ester (carbonyl) groups may contribute to improved local order and stabilization of the surface layer, allowing for more effective exposure of hydrophobic segments at the fiber–air interface. These cooperative effects help minimize the surface energy of the treated fabric, resulting in enhanced water repellency. Thanks to the presence of an ester group in the silane structure (S4), it was possible to achieve highly hydrophobic cotton fabrics with a water contact angle of 142°, using a simple, fluorine-free alkyl silane.

The durability of the modification applied to the cotton fabrics was evaluated through repeated washing cycles. The results showed only minimal changes in water repellency after multiple laundering tests, confirming the robustness of the silane treatments. Importantly, the hydrophobic properties remained stable throughout the washing procedure, indicating excellent resistance to laundering and demonstrating the practical suitability of the developed silane coatings for long-term use in textile applications.

### 2.6. Oleophobic Properties of Modified Cotton Fabrics

**Figure 5:**
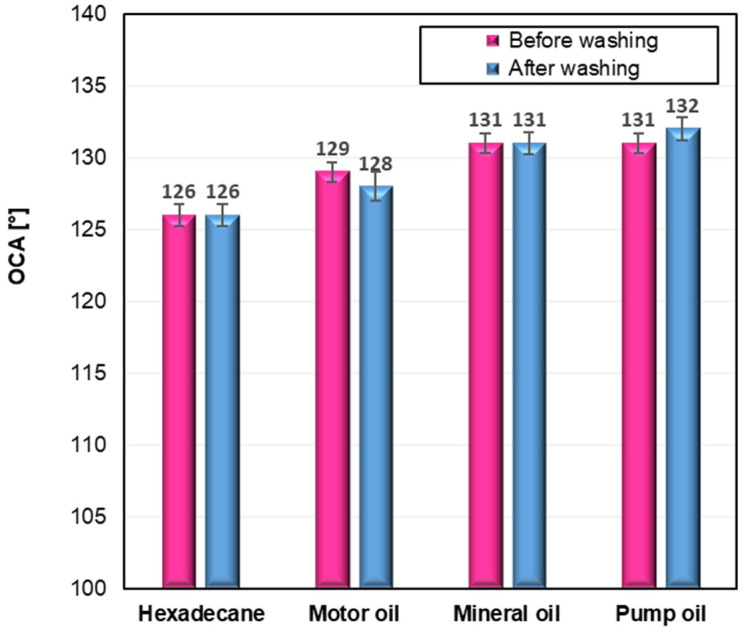
Oil contact angle (OCA) of modified cotton fabrics (CS2) before and after washing. The corresponding standard deviations for samples are as follows: Hexadecane 126 ± 0.8, 126 ± 0.8; Motor oil 129 ± 0.7, 128 ± 7; Mineral oil 131 ± 0.7, 131 ± 0.8; Pump oil 131 ± 0.7, 132 ± 0.8.

To evaluate the oleophobic behaviour of the modified cotton fabrics, oil contact angles (OCA) were measured using the same video-based goniometer system that was employed for the WCA determinations. Each measurement was repeated five times to ensure accuracy and reproducibility. The oleophobic performance of the samples was assessed using various test oils, and the obtained OCA values for the fabric modified with the C6-fluorinated silane (CS2) are presented in Figure 5.

**Figure 6:**
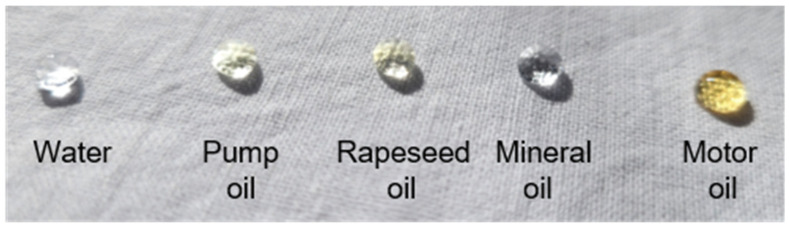
Photograph of water and various oils drops on CS2 fabric.

The oleophobic properties of the modified cotton fabrics were evaluated by measuring oil contact angles (OCA) using several representative oils. As shown in Figure 5, only the fabric modified with the C6-perfluoroalkyl silane (CS2) demonstrated clear oleophobic behavior, with OCA values in the range of 126–132°, which remained virtually unchanged after ten washing cycles. These results confirm the excellent durability and resistance to oil penetration achieved through this modification. In contrast, the remaining samples (CS1, CS3, and CS4) did not exhibit measurable oleophobicity, and therefore OCA determination was not possible for those fabrics. The stability of the oleophobic response before and after washing further highlights the robustness of the CS2 coating and its suitability for long-term practical use. Figure 6 presents an image illustrating the behavior of water and various oil droplets on the surface of the CS2-treated fabric, clearly demonstrating its dual hydrophobic and oleophobic performance.

### 2.7. Water Absorption Tests

**Figure 7:**
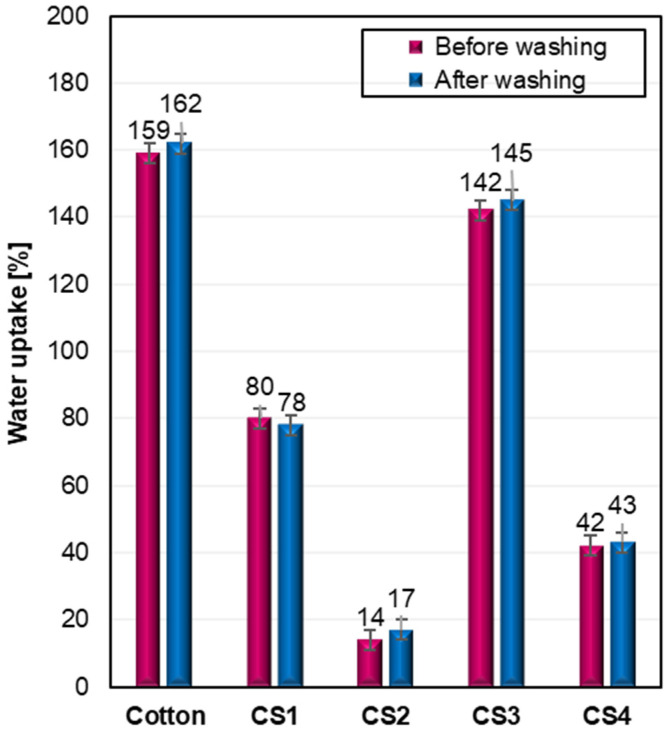
Water uptake of raw and modified cotton fabrics (CS1–CS4) before and after washing.

The water absorption capacity of the cotton fabrics was evaluated both before and after repeated washing, as shown in Figure 7, to assess the effectiveness and durability of the applied silane modifications.

As expected, the unmodified fabric exhibited the highest absorption values (159% before washing and 162% after), reflecting the inherent hydrophilic nature of cellulose. In contrast, all modified fabrics displayed significantly reduced water uptake, confirming the hydrophobic character imparted by the applied silanes. Among them, sample CS2 (bearing a C6 perfluorinated chain) showed the lowest absorption values—14% before and 17% after washing, demonstrating the highest resistance to water penetration. Sample CS4, containing a long non-fluorinated alkyl chain C12, also effectively limited water uptake (42–43%), indicating that high hydrophobicity can be achieved even without fluorine, provided the hydrophobic chain is sufficiently long. Interestingly, cotton CS1, which features a branched fluorinated substituent, exhibited notably higher absorption (80–78%) than CS2, suggesting that molecular geometry and the length of the fluorinated chain plays a significant role, and that branching may hinder optimal surface orientation. CS3, functionalized with a benzyl group, demonstrated poor performance among the modified samples (142–145%).

The photos in Table 4 show the results of water drop absorption of modified and washed cotton fabrics (CS1W–CS4W). In each case, 20 µL of dyed water drops were applied on the fabrics.

As can be seen, among the four types of modifications tested, the fabrics modified with silanes containing a branched fluorinated chain (blue drop, CS1W) and a benzyl substituent (green drop, CS3W) did not show stable water droplets on their surfaces—the drops were absorbed within a few minutes after application. These results suggest that droplet stability is not solely dependent on the presence of fluorine or high contact angle values, but is also influenced by the molecular structure. This observation aligns with earlier reports which noted that high water contact angles do not necessarily correlate with prolonged droplet stability, a critical factor in assessing real-world performance of hydrophobic textiles [44]. Therefore, droplet absorption time should be considered an essential complementary parameter to contact angle measurements when characterizing surface hydrophobicity, particularly for applications where prolonged exposure to moisture may occur.

On the other hand, fabrics CS2W and CS4W did not absorb water during the 30 min tests and show highly hydrophobicity. Water did not diffuse into the fabrics, but remained on the surfaces. This is due to the fact that fabrics were covered by C6 perfluorinated chain (orange drop, CS2W) or long hydrophobic alkyl chain (pink drop, CS4W). The water droplets remained stable on the surfaces for at least 30 min, indicating excellent water repellency and confirming the formation of highly hydrophobic surfaces. Only a decrease in the size over time was observed due to the evaporation of water.

**Figure 8:**
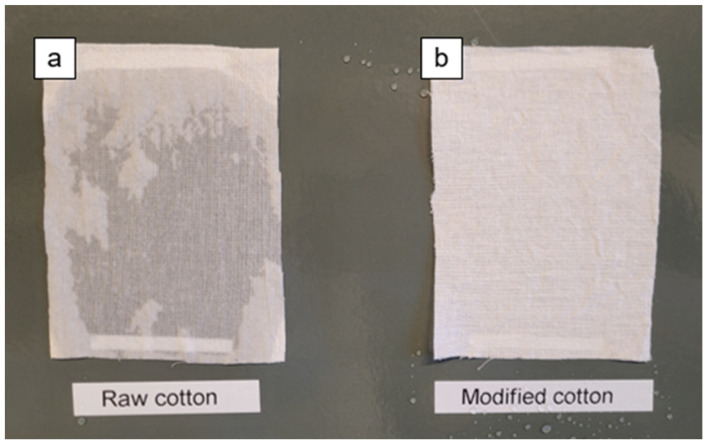
Photograph of raw cotton (**a**) and modified cotton fabric (CS2) (**b**) after pouring a stream of water.

During the final test, two cotton fabrics (unmodified and modified) were exposed to a water jet, and the wettability of the materials was observed, as shown in Figure 8. The fabric on the left (a) was raw, while the right (b) was treated with the S2 derivative. It was observed that modified cotton fabric was not wet, indicating the excellent water resistant ability of the coatings. The videos available in the Supplementary Information (Videos B.1–B.3) shows real-time observation of the excellent water resistant property of the coating upon pouring a stream of water onto it.

## 3. Discussion

Excellent results were obtained for the cotton sample CS2, which showed a water contact angle (WCA) of 152° and an oil contact angle (OCA) of 126° (hexadecane). These values are particularly impressive considering that the applied silane contained a relatively short perfluoroalkyl chain (six fluorinated carbon atoms), exhibited a low add-on value, required only a short impregnation time (30 min), and demonstrated excellent washing durability. Moreover, the modification of cotton with silane S2 led to a significant reduction in water absorption—by approximately 91% compared to unmodified cotton—confirming the formation of a dense and highly effective hydrophobic barrier. For comparison, Li et al. [38] achieved similar or slightly higher contact angles (WCA of 163.5° and OCA of 120.1°) by grafting fluorinated POSS via silane coupling and thiol–ene click reactions. However, their approach involved the use of longer perfluoroalkyl chains (eight fluorinated carbon atoms), expensive POSS-based precursors, and more complex synthesis conditions. In the same study, a reference sample modified only with 1*H*,1*H*,2*H*,2*H*-perfluorodecyltrimethoxysilane (containing an eight-carbon perfluoroalkyl chain) exhibited lower contact angles (WCA of 142.4° and OCA of 78.0°), confirming the efficiency of our simpler and more sustainable modification route, even at low silane deposition levels. Ferrero and Periolatto have obtained very high water and oil contact angles (169°) using 1*H*,1*H*,2*H*,2*H*-perfluorooctyltriethoxysilane (silane containing a chain of six perfluorinated carbon atoms) or Fluorolink S10 (commercial product) in combination with a sol–gel process. However, their method required a 24 h immersion process and relatively high silane concentrations to maintain water and oil repellency after washing. Furthermore, despite the high contact angle values, water droplets placed on the surface were eventually absorbed, indicating that the surface did not fully prevent wetting over time [45]. In contrast, in our study, water droplets remained on the surface of CS2-treated cotton for at least 30 min without any absorption, confirming the formation of a highly stable hydrophobic layer with superior dynamic performance.

The significance of this work lies not only in achieving high WCA and OCA values but also in demonstrating the effectiveness of a simpler and more sustainable surface treatment process. Compared to other reports, our method involves milder conditions (room-temperature hydrolysis, short impregnation time, and low silane concentration), while still producing hydrophobic and oleophobic coatings with excellent durability. Moreover, in contrast to other studies, we achieved comparable performance using a short-chain fluorinated silane (chain of six perfluorinated carbon atoms), which is considered less bioaccumulative and toxic [36].

A limitation of the present study is the continued use of fluorinated compounds. Although C6-based fluorosilanes are generally recognized as safer alternatives to long-chain PFAS (C8 and above), they are not fully environmentally benign. Short-chain perfluoroalkyl substances exhibit lower bioaccumulation but remain environmentally persistent and difficult to degrade, and their long-term ecotoxicological impact is still under investigation. As highlighted in recent reviews, even C6 derivatives may contribute to PFAS accumulation in water and soil ecosystems and therefore should be used cautiously [18,46]. For this reason, we also proposed a fluorine-free alternative based on a long-chain alkyl silane (C12), which yielded high WCA values (140° before and 143° after washing) and excellent droplet stability, proving that effective hydrophobicity can be achieved without fluorinated moieties.

The developed modification approach shows strong potential for practical applications in technical textiles, protective clothing, outdoor fabrics, and upholstery materials, where durable water and oil repellency combined with good washing resistance are required. Additionally, the simplicity and mildness of the process make it suitable for large-scale textile finishing under environmentally responsible conditions.

In summary, this work contributes to the ongoing efforts to develop effective and sustainable hydrophobic textile coatings. Recent literature demonstrates a clear trend towards balancing high performance with environmental responsibility, with a growing focus on short-chain fluorinated and fluorine-free alternatives. However, there are still conflicting reports about how durable and effective these coatings are, especially after repeated washing—some studies show that repellency quickly decreases, while others demonstrate that the coatings remain effective over a long period. These discrepancies highlight the influence of molecular structure, deposition technique, and post-treatment conditions on coating stability. A significant research gap persists in the development of scalable, eco-friendly surface modification methods that simultaneously achieve high repellency, durability, and minimal environmental impact, particularly for oleophobic systems. Our findings support the potential of short-chain fluorinated silanes as a more sustainable option, but further research is needed to optimize fluorine-free alternatives and to establish standardized testing protocols for long-term performance and environmental safety [18,46].

## 4. Materials and Methods

### 4.1. Materials

Silanes were synthesized through the process outlined in our previous paper [43]. Acetic acid (99,5%) and tetrahydrofuran (99%) were purchased from Sigma Aldrich. Dodecanethiol (98%) was purchased from Acros Organics. Vinyltrimethoxysilane (98%) was purchased from Fluorochem. 2,2-Dimethoxy-2-phenylacetophenone (DMPA) and CDCl_3_ were purchased from Aldrich. Reagents were used without further purification. A bleached woven cotton fabric supplied by the Textile Factory in Łódź (Poland) was used as the textile substrate. The fabric was produced in a plain weave, with an areal density of 145 g/m^2^. The thread density was 254 warp threads/dm and 228 weft threads/dm, and the yarn linear density was 26.71 tex (warp) and 28.93 tex (weft). According to the quality report, the textile exhibited a 67% whiteness degree (Stephansen method) and showed no starch residue.

### 4.2. Analytical Methods

#### 4.2.1. NMR Analysis

^1^H NMR, ^13^C NMR and ^29^Si NMR spectra were recorded using a Bruker Avance NEO 400 MHz spectrometer, Rheinstetten, Germany. All measurements were performed at room temperature using CDCl_3_ as the solvent. Spectra were processed with standard Bruker software, TopSpin 4.1.3.

#### 4.2.2. Contact Angle Measurements

Static water (WCA) and oil contact angle (OCA) measurements were performed using an automatic video contact-angle system (Krüss GmbH, model DSA 100 Expert, Hamburg, Germany). The apparatus was equipped with DAS4 2.0 software for automatic positioning of the measuring table (*x*, *y*, *z* axes), a four-channel dosing unit, and a 780 × 580-pixel camera with adjustable focus, contrast, exposure time, and illuminance. For WCA measurements, 5 μL droplets of deionized water were used. For OCA measurements, 5 μL droplets of four representative oils—hexadecane, motor oil, mineral oil, and pump oil—were applied to the fabric surface. All contact angles were determined using the circle (tangent) fit model at 0° camera tilt. Each reported WCA or OCA value represents the average of five independent measurements, recorded at different locations on the sample to ensure reproducibility and account for any local surface variations.

#### 4.2.3. SEM-EDS Analysis

The morphology and surface elemental composition of the fabrics were examined using a Hitachi SU-3500 scanning electron microscope (SEM) equipped with an energy-dispersive X-ray spectroscopy (EDS) detector, Tokyo, Japan. The analyses were performed at an accelerating voltage of 15 kV, with gold-coated samples to enhance surface conductivity. The measurements were carried out in Poznań, Poland.

#### 4.2.4. Elasticity Measurement

The test followed the ASTM D 1388. Samples were cut to the size of 2.5 cm × 20 cm. Samples were placed on a horizontal platform and slid until their leading edges projected from the edge to form a 41.5° angle with the horizontal. The overhang length (O) was recorded. Tests were done with 5 samples tested for each fabric type.

c = O/2

G= 1.421·10^−5^·W·c^3^ [µjoule/m]

c = bending length (mm), W = fabric unit mass (g/m^2^)

### 4.3. Synthesis of Silanes S1–S5

For cotton fabrics modification five silanes were used, differing in type of functional groups and linkers. The structural formulas of the silanes used are shown in Table 1. Silanes S1–S4 were synthesized according to our previous paper [43], namely via thiol-Michael addition reaction between thiol, i.e., 3-mercaptopropyltrimethoxysilane and acrylates with fluoroalkyl (S1) or C12 alkyl group (S4), or methacrylates with fluoroalkyl (S2) or benzyl group (S3) in the presence of a catalyst (dimethylphenylphosphine, DMPP).

**Figure 9:**
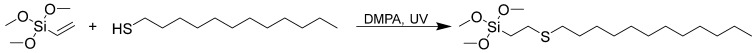
Synthesis of silane S5 via thiol-ene reaction.

Silane S5 was synthesized by thiol-ene process between vinyltrimethoxysilane and 1-dodecanethiol in the presence of a photoinitiator (2,2-dimethoxy-2-phenylacetophenone, DMPA) and UV light, according to Figure 9.

A round-bottomed flask equipped with a magnetic stirrer was loaded with vinyltrimethoxysilane (2.25 g, 2.19 mmol), 1-dodecanethiol (2.94 g, 2.19 mmol), and photoinitiator 2,2-dimethoxy-2-phenylacetophenone (DMPA, 36.5 mg, 0.285 mmol). The reaction mixture was stirred for 2 min and then irradiated with the use of a medium pressure mercury lamp of 400 W and generating wavelengths from the range 280–600 nm for 1 h at room temperature. After a few minutes of irradiation, slight heating of the reaction mixture was observed. The product was purified by “trap-to-trap” distillation. The isolation yield of the synthesis was 81% (4.26 g).

The structure of the synthesized compound was confirmed by NMR spectroscopy (^1^H, ^13^C and ^29^Si NMR spectra are included in Supplementary Information (Figures S1–S3)).

**^1^H NMR** (400 MHz, CDCl_3_) δ(ppm): 3.50 (s, 9H, Si(OCH_3_)_3_); 2.58–2.50 (m, 2H, SCH_2_); 2.47–2.40 (m, 2H, SCH_2_); 1.50 (tt, *J* = 8.3, 7.3 Hz, 2H, CH_2_); 1.36–1.27 (m, 2H, CH_2_), 1.27–1.10 (m, 16H, CH_2_); 0.96–0.88 (m, 2H, SiCH_2_); 0.83–0.77 (m, 3H, CH_3_). **^13^C NMR** (101 MHz, CDCl_3_) δ(ppm): 50.50; 31.93; 31.91; 29.67; 29.65; 29.62; 29.58; 29.56; 29.36; 29.28; 28.98; 25.99; 22.69; 14.07; 10.58. **^29^Si NMR** (80 MHz, CDCl_3_) δ(ppm): −44.76.

### 4.4. Modification of Cotton Fabrics

The modification of cotton fabrics was carried out in two main steps: hydrolysis of silanes and fabric treatment. Silanes were hydrolyzed in a round-bottom flask equipped with a reflux condenser and a magnetic stirrer using a solution containing 5 wt.% silane, 5 wt.% acetic acid, 5 wt.% water, and 85 wt.% tetrahydrofuran (THF). The mixture was stirred at room temperature for 1 h to complete the hydrolysis process. Bleached cotton fabrics were then cut into 8 × 11 cm samples and immersed in the hydrolyzed silane solutions for 30 min at room temperature. After immersion, the excess solution was gently removed using paper towels, and the samples were dried at 80 °C for 1 h. Finally, the modified fabrics were cured at 130 °C for 3 min to promote condensation and the formation of a stable siloxane network on the fiber surface.

### 4.5. Washing

The modified fabric samples were subjected to repeated laundering based on the PN-EN ISO 105-C06:2010 standard [47], which outlines methods for evaluating color fastness under household and industrial washing conditions. Each washing cycle was performed at 40 °C for a duration of 60 min in the presence of detergent, followed by intensive rinsing. To assess the durability of the surface treatment, the entire process was repeated ten times.

### 4.6. Add-On Value

To determine add-on value, cotton samples were weighed before and after modification and add-on percentage was calculated according to the Formula (1).

(1) $$Add–on=\frac{m_{1}-m_{0}}{\;m_{0}}\times 100$$

where *Add-on*—the add-on value [%], *m*_0_—the weight of the cotton fabric before modification [g], *m*_1_—the weight of the cotton fabric after modification [g].

### 4.7. Evaluation of Hydrophobic and Oleophobic Properties

The hydrophobic behaviour of the treated cotton fabric samples was assessed using a combination of contact angle measurements and water absorption tests. Static water contact angles were determined with the aid of a high-precision video-based goniometer (Krüss DSA 100 Expert). For each sample, a 5 µL droplet of distilled water was gently placed on the fabric surface, and the contact angle was calculated from image sequences recorded during droplet deposition. Each reported value represents the average of five independent measurements to ensure accuracy and reproducibility. The oleophobic behaviour of the treated cotton fabric samples was assessed using the same method and apparatus and hexadecane as a test liquid. To examine the absorbency of individual water droplets, a single water droplet was applied to the surface of each fabric sample, and the time required for complete absorption was recorded using a stopwatch to a maximum of 1800 s. This method provides a qualitative indication of surface wettability. Additionally, the overall water uptake (A, expressed as weight percent) was determined following the procedure described by Mahltig and Böttcher. Fabric samples measuring 11 × 8 cm were mounted in a clamp and fully immersed into a beaker filled with distilled water. To prevent the entrapment of air beneath the samples, the textiles were introduced at a vertical position. After one minute of immersion, the entire clamp assembly was removed, and the samples were allowed to hang freely in a horizontal support for three minutes to allow excess water to drain. The fabrics were then reweighed and the water uptake was calculated accordingly (1). This procedure was conducted in accordance with the DIN 53923 standard [48]. The measurements for each sample were repeated five times.

## 5. Conclusions

In this work, five organosilicon derivatives were synthesized and applied to cotton fabrics using a mild sol–gel silanization process to generate durable hydrophobic and oleophobic properties. Each silane incorporated an alkoxysilyl group enabling covalent bonding to cellulose, as well as a hydrophobic or oleophobic functional group responsible for lowering the surface energy. The ester linker formed via the thiol–Michael addition played a role in molecular organization, supporting dipolar interactions between carbonyl groups and enhancing the orientation of functional substituents on the fiber surface.

Among the tested systems, the silane containing a C6 perfluoroalkyl chain (CS2) demonstrated the best performance, achieving a WCA of 152° before washing and 153° after washing, and an OCA of 126° (hexadecane) under both conditions. This sample also showed the lowest water absorption (14% before and 17% after washing; 91% reduction versus raw cotton) and maintained droplet stability for more than 30 min. The fluorine-free analogue bearing a long C12 alkyl chain (CS4) also yielded high hydrophobicity (WCA 140–143°), confirming that effective water repellency can be achieved without using perfluorinated groups.

The cotton fabrics were modified using a simple and fast silanization procedure. A 5 wt.% silane solution was hydrolyzed for 1 h at room temperature and then applied to the fabric by a 30 min immersion, followed by drying at 80 °C for 1 h and curing at 130 °C for 3 min. These mild and easily applicable conditions yielded uniform coatings with low add-on values and very good washing durability.

SEM–EDS analyses confirmed the presence and persistence of the silane coatings after ten washing cycles. Taken together, the results demonstrate that both short-chain fluorinated silanes and long-chain alkyl silanes can impart durable water-repellent—and in the case of CS2, oleophobic—properties using a simple, low-energy and environmentally responsible finishing process.

The developed modification strategy shows strong potential for technical textiles, outdoor garments, protective fabrics, and upholstery applications requiring long-lasting liquid repellency. Future research should focus on fully fluorine-free or bio-based alternatives, as well as evaluating the mechanical durability and long-term environmental stability of the coatings.

## Figures and Tables

**Table 1:** Structure of silanes S1–S5 used for modification of cotton fabrics.

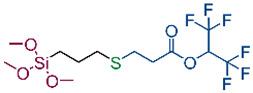	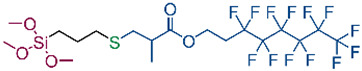
**S1**	**S2**
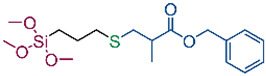	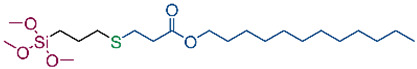
**S3**	**S4**
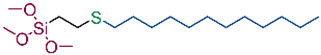	
**S5**	

**Table 2:** Add-on and SEM-EDS results of raw cotton (Cotton) and modified cotton fabrics (CS1–CS4).

Sample Code	Add-On [%]		SEM-EDS [wt.%]									
			Before Washing					After Washing				
	Before Washing	After Washing	C	O	Si	S	F	C	O	Si	S	F
Cotton	1.4 (±0.2)	1.3 (±0.2)	34.1	64.3	-	-	-	34.1	64.8	-	-	-
CS1	2.1 (±0.2)	2.1 (±0.2)	33.9	60.9	0.7	0.4	0.2	33.3	65.3	0.8	0.4	0.2
CS2	2.9 (±0.2)	2.8 (±0.2)	33.7	62.6	0.9	0.6	3.4	32.1	62.9	0.8	0.6	3.2
CS3	3.6 (±0.2)	3.6 (±0.2)	34.3	64.3	1.5	1.4	-	33.8	63.4	1.3	1.4	-
CS4	1.4 (±0.2)	1.3 (±0.2)	33.0	64.3	1.3	1.2	-	35.1	61.8	1.4	1.2	-

**Table 3:** Bending length and fexural rigidity of pure cotton and modified cotton fabrics.

	Before Washing		After Washing	
Symbol	Bending Length [mm]	Flexural rigidity [µJ/m]	Bending Length [mm]	Flexural rigidity [µJ/m]
**Cotton**	15	7	15	7
**CS1**	14.5	6.3	14.5	6.3
**CS2**	14	5.7	14	5.7
**CS3**	14.5	6.3	14.5	6.3
**CS4**	14.5	6.3	14.5	6.3

**Table 4:** Images of dyed water drops (20 µL) on modified cotton fabrics (after washing) over time.

Sample Code	Time				
	1 s	1 min	5 min	15 min	30 min
CS1W	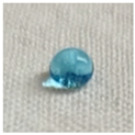	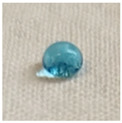	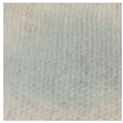	-	-
CS2W	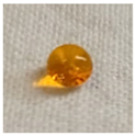	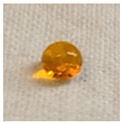	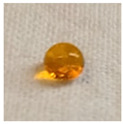	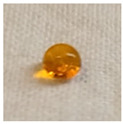	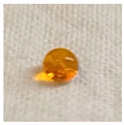
CS3W	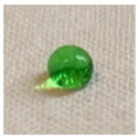	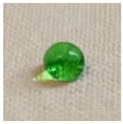	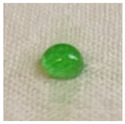	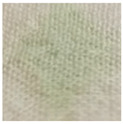	-
CS4W	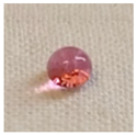	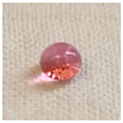	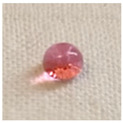	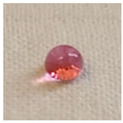	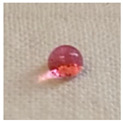

## Data Availability

The original contributions presented in this study are included in the article/Supplementary Material. Further inquiries can be directed to the corresponding author(s).

## Supplementary Materials

The following supporting information can be downloaded at: https://www.mdpi.com/article/10.3390/ijms262311374/s1.
