# Supplementary material for: Silanization of Cotton Fabric to Obtain Durable Hydrophobic and Oleophobic Materials
Source: Int J Mol Sci. 2025 Nov 25;26(23):11374. doi: 10.3390/ijms262311374 (PMC12692723; doi:10.3390/ijms262311374)
Supplement: Supplementary file 1 [file ijms-26-11374-s001.zip › ESI MP.pdf]

SUPPORTING INFORMATION

Silanization of Cotton Fabric To Obtain Durable Hydrophobic and Oleophobic Materials

Anna Szymańska [1], Marcin Przybylak [1,*], Agnieszka Przybylska [2] and Hieronim Maciejewski [1,2]

[1] Poznań Science and Technology Park, Adam Mickiewicz University Foundation, Rubież 46, 61-612 Poznań, Poland; anna.szymanska@ppnt.poznan.pl, hieronim.maciejewski@ppnt.poznan.pl
[2] Adam Mickiewicz University, Uniwersytetu Poznańskiego 8, 61-614 Poznań, Poland; agnieszka.przybylska@amu.edu.pl,
* Correspondence: marcin.przybylak@ppnt.poznan.pl ; Tel.: +48 61 827 97 54

Table of contents

NMR spectra of silane S5 3

NMR spectra of silane S5

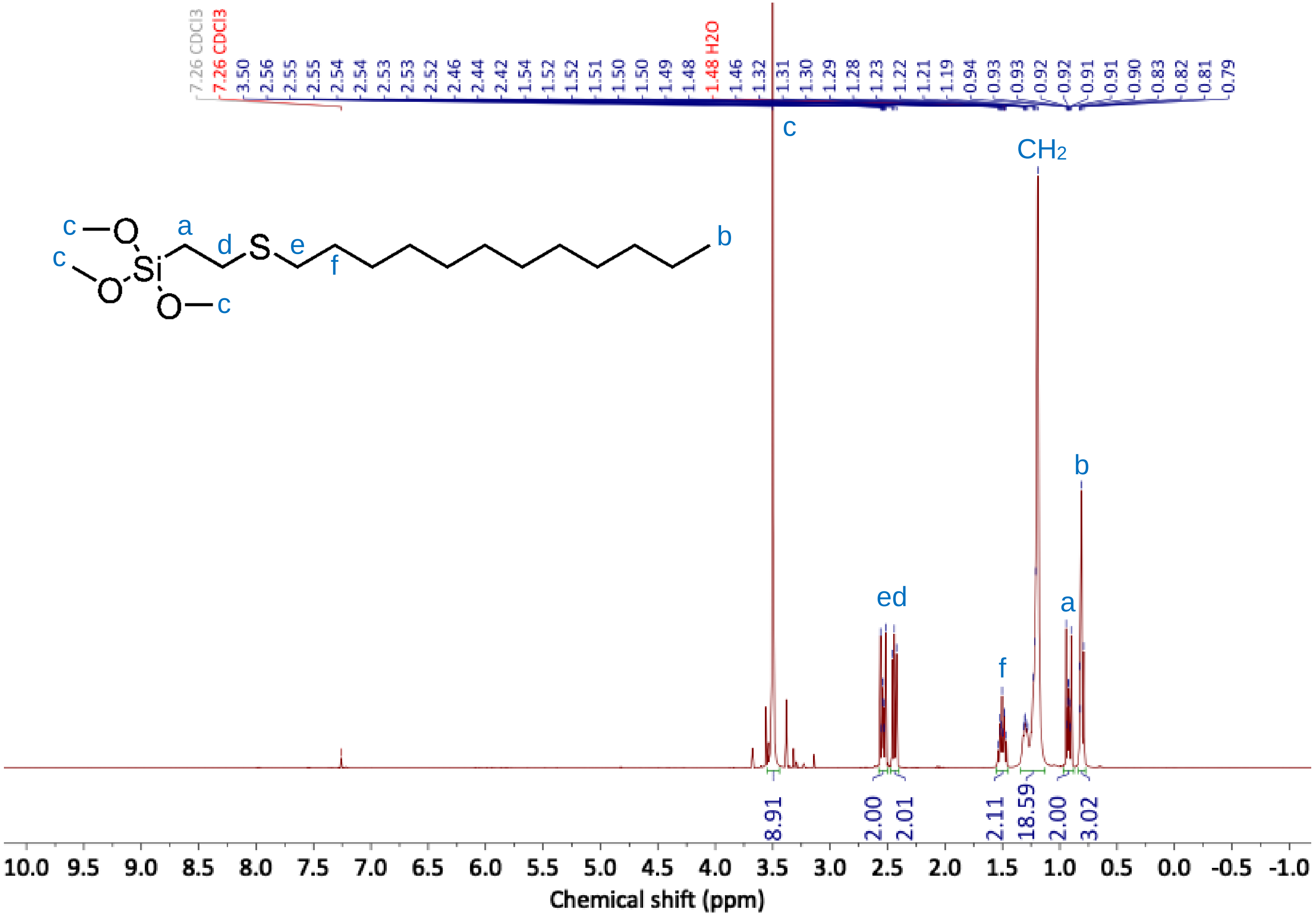

Figure S1 The ^{1}H NMR spectra of S5.

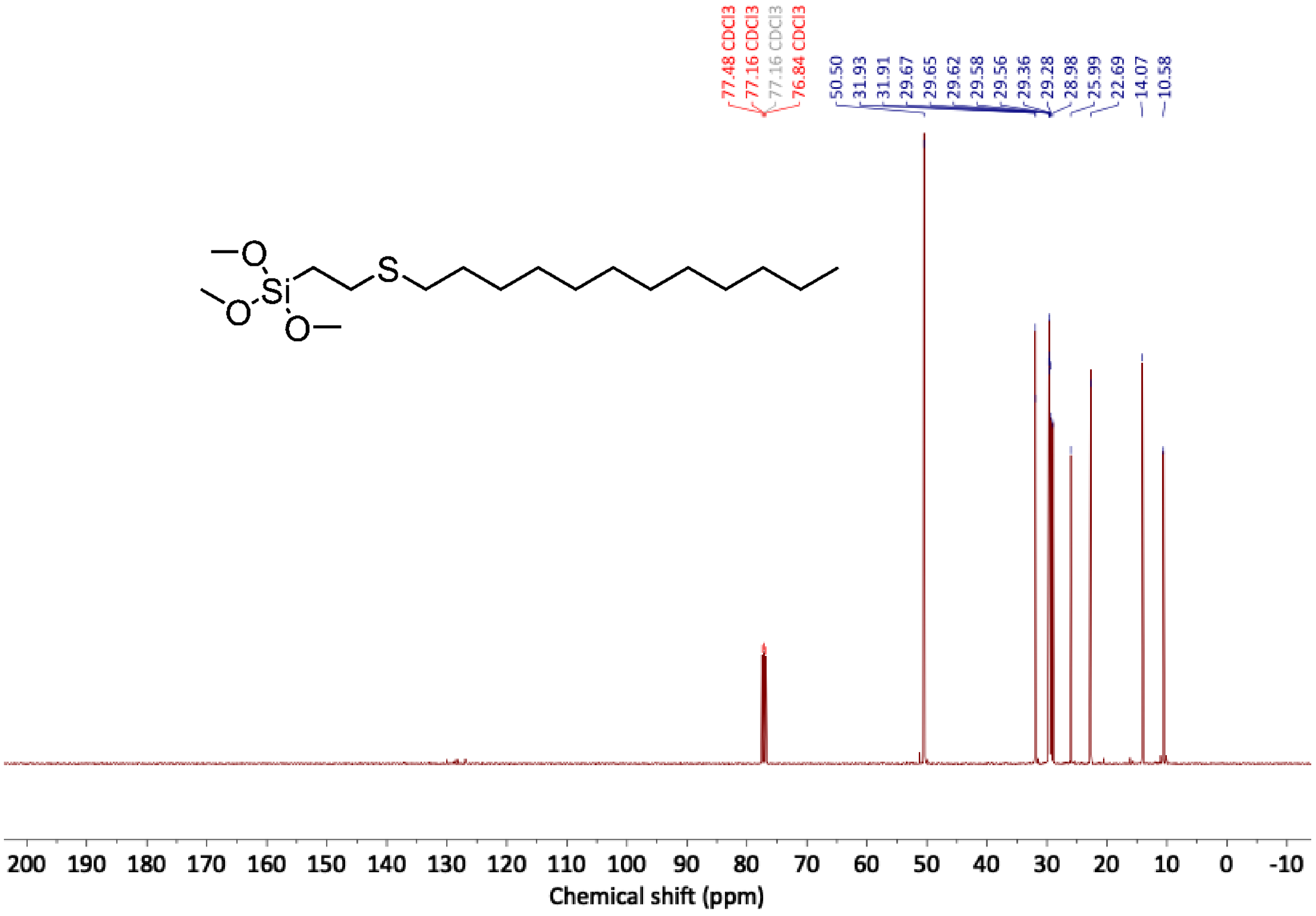

Figure S2 The ^{13}C NMR spectra of S5.

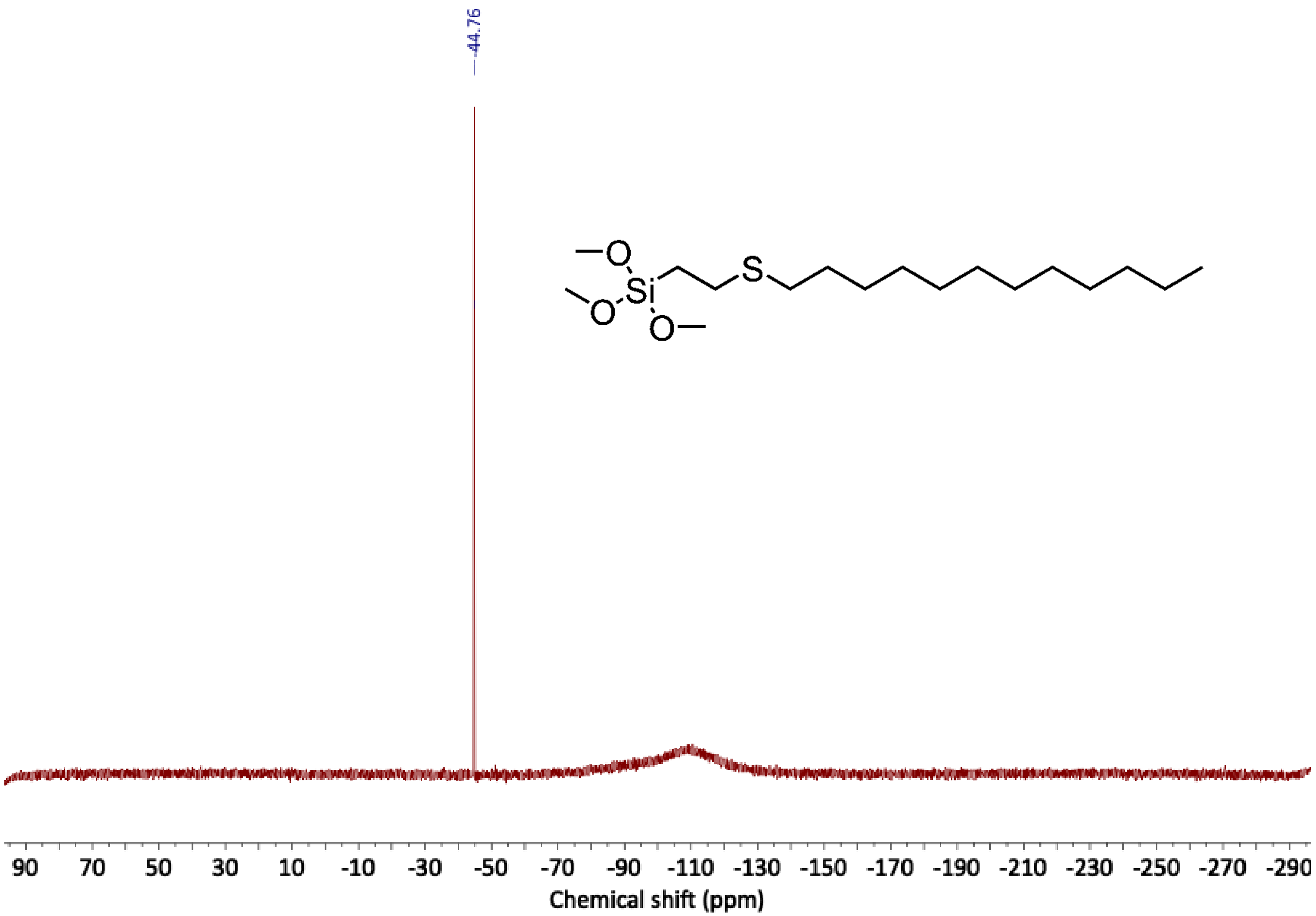

Figure S3 The ^{29}Si NMR spectra of S5.
